# Iron-Based Metal-Organic Frameworks as a Theranostic Carrier for Local Tuberculosis Therapy

**DOI:** 10.1007/s11095-018-2425-2

**Published:** 2018-05-18

**Authors:** Gabriela Wyszogrodzka, Przemysław Dorożyński, Barbara Gil, Wieslaw J. Roth, Maciej Strzempek, Bartosz Marszałek, Władysław P. Węglarz, Elżbieta Menaszek, Weronika Strzempek, Piotr Kulinowski

**Affiliations:** 1 Faculty of Pharmacy, Department of Pharmacobiology, Jagiellonian University Medical College, Medyczna 9, 30-068 Kraków, Poland; 2 Pharmaceutical Research Institute, Rydygiera 8, 01-793 Warszawa, Poland; 3 Faculty of Chemistry, Jagiellonian University in Kraków, Gronostajowa 2, 30-387 Kraków, Poland; 4 Department of Magnetic Resonance Imaging, Institute of Nuclear Physics, Polish Academy of Sciences, Radzikowskiego 152, 31-342 Kraków, Poland; 5 Faculty of Pharmacy, Department of Cytobiology, Jagiellonian University Medical College, Medyczna 9, 30-068 Kraków, Poland; 6 Faculty of Mathematics, Physics and Technical Science, Institute of Technology, Pedagogical University of Cracow, Podchorążych 2, 30-084 Kraków, Poland

**Keywords:** iron metal-organic framework (MOF), MRI contrast agent, theranostic system, tuberculosis treatment, inhaled dosage forms

## Abstract

**Purpose:**

The purpose of the study was initial evaluation of applicability of metal organic framework (MOF) Fe-MIL-101-NH_2_ as a theranostic carrier of antituberculous drug in terms of its functionality, i.e. drug loading, drug dissolution, magnetic resonance imaging (MRI) contrast and cytotoxic safety.

**Methods:**

Fe-MIL-101-NH_2_ was characterized using X-ray powder diffraction, FTIR spectrometry and scanning electron microscopy. The particle size analysis was determined using laser diffraction. Magnetic resonance relaxometry and MRI were carried out on phantoms of the MOF system suspended in polymer solution. Drug dissolution studies were conducted using Franz cells. For MOF cytotoxicity, commercially available fibroblasts L929 were cultured in Eagle’s Minimum Essential Medium supplemented with 10% fetal bovine serum.

**Results:**

MOF particles were loaded with 12% of isoniazid. The particle size (3.37–6.45 μm) depended on the micronization method used. The proposed drug delivery system can also serve as the MRI contrast agent. The drug dissolution showed extended release of isoniazid. MOF particles accumulated in the L929 fibroblast cytoplasmic area, suggesting MOF release the drug inside the cells. The cytotoxicity confirmed safety of MOF system.

**Conclusions:**

The application of MOF for extended release inhalable system proposes the novel strategy for delivery of standard antimycobacterial agents combined with monitoring of their distribution within the lung tissue.

## Introduction

The use of nanotechnology for medical applications is rapidly growing and is very promising in various branches of applied science (1–4). Nanoparticles (NPs) are used as diagnostic imaging agents or as drug delivery platforms, providing targeted or tissue-selective therapy, which may increase efficiency and decrease the side effects of drugs. It is also possible to combine these two functions in one particle by the design and preparation of dual-purpose nanomaterials, functioning as both diagnostic medical devices and drug delivery systems (5,6). This concept of fusing diagnostics and therapy has been proposed in 2002 and called theranostics (7). Theranostic agents have been defined as “integrated nanotherapeutic systems, which can diagnose, deliver targeted therapy, and monitor the response to the therapy” (6). This integrated approach offers great opportunities in the development of personalized medicine It allows for monitoring the drug release, its biodistribution and accumulation at the target site, dose adjustment to individual patients and finally, monitoring the course of a disease (5,8,9).

Freund et al. has proposed the term “atom economy” which focuses on the design of highly active materials possessing many functionalities that work together to serve a specific purpose (10). Metal-Organic Frameworks (MOFs) are excellent example to illustrate this concept and have the potential to emerge as next-generation drug delivery systems (DDS). MOFs may be of interest as carriers for theranostics, being porous structures built from inorganic nodes, which are single ions or clusters of ions, joined together by organic linkers. Such design allow to gain control over the framework architecture and, even more importantly, the pore chemistry, enabling targeted functionalization for nanomedical applications (11). Thanks to their porous structure, MOFs seem to be promising drug vehicles with potential high drug loading (12–14). The control of guest release profiles can be gained by the choice of the type of functional group of the linker and tuning of the pore size (15).

In the case of MOF application for drug delivery, they should exhibit stability under physiological condition, minimal toxicity, biodegradability and as biocompatibility for both metal and bridging linker ligand. MOFs as components of drug delivery system are discussed in the context of other nanoparticulate carriers (mesoporous silica, dendrimers) in the work by Wuttke et al. (16). Safety of MOF as drug delivery nanomaterials varies strongly with effector cell type. Therefore, it is necessary to evaluate their nanosafety regarding particular application and involved cell type. Wuttke et al. (11) discuss the effects of MIL-101(Cr) and MIL-101(Fe) on human endothelial and mouse lung cells, a first line of defense upon systemic blood-mediated and local lung-specific applications of nanoparticles.

Magnetic resonance imaging (MRI) has become a powerful tool in medicine for non-invasive imaging of the internal structure and functions of living organisms as well as local properties of tissues (17). Magnetic nanoparticles may be applied as contrasting agents providing either negative (T_2_-weighted) or positive imaging contrast (T_1_-weighted) (18). A potential MRI contrasting agent has to fulfill several requirements related to tolerance, safety, toxicity, stability, osmolarity, viscosity, biodistribution, elimination, and metabolism (19).

Embedding paramagnetic cations (Gd^3+^, Mn^2+^, Fe^3+^) in MOF structure make them possible to be used as MRI contrast agents. Among them, iron is the best option from a toxicological point of view. Nanoscale iron MOFs (MIL-53, MIL-88A, MIL-88Bt, MIL-89, MIL-100 and MIL-101_NH_2_), with engineered cores and surfaces, have been shown to be able to serve as drug carrier and magnetic resonance contrast agent according to good ability for modification of relaxivities (20).

Polymeric surface allows for the MOF structures modification to implement properties such as increased chemical and colloidal stability which enhance the cellular uptake, or dye-labeling, which enables for example, the investigation of nanoparticle uptake into tumor cells by fluorescence microscopy (21). It has been proved, that coated iron MOFs can retain their MRI contrast properties. MOF NPs are frequently coated in order to prevent leakage of the drug before they reach the target (e.g. exosome-coated MIL-88A, liposome coated MIL-88A) (21,22). MOFs, including iron MOFs, can be (multi)functionalized (example can be found in the work of Roeder et al. 2017 (21)) – molecular units can be anchored on the outer surface of MOFs.

MOFs can be used as stimuliresponsive DDS for cancer therapy (targeted chemotherapy, gene therapy). Recent studies also shown that it is possible to develop MOF based delivery systems for photodynamic therapy (PDT) of cancer (23).

While theranostics has been intended mainly for cancer treatments, there are numerous other therapeutic targets for which the effectiveness of therapy could be increased by local drug delivery and monitoring of its distribution. Among them tuberculosis (TB) seems to be especially important.

TB is one of the top three infectious diseases – together with HIV and malaria – causing morbidity and death worldwide. According to the World Health Organization (WHO) estimations, about 30% of the world’s population is infected with *Mycobacterium tuberculosis* (MTB). Every year, about 10 million of new cases are registered and about 1.5 to 2 million of deaths are caused by TB, according to the report by the WHO agency (24). TB infections frequently become multidrug-resistant, since the conventional treatment protocol is based on an antibiotic therapy carried out over periods of 6 to 9 months (25).

Isoniazid (Isonicotinic Acid Hydrazide, INH) is particularly suitable for use as a model drug in theranostic drug delivery because it is an antibiotic used as a first-line agent in the prevention and treatment of both latent and active tuberculosis. It is effective against mycobacteria, especially *Mycobacterium tuberculosis* since it inhibits biosynthesis of the mycolic acid. Oral administration of INH and long-term therapy causes several serious side effects, which could even force treatment discontinuation. INH is known to cause hepatitis (26), hepatic injury and neuropsychiatric disturbances (27) including, among others, uncontrollable seizures (28) or polyneuropathy (29). The conventional oral route of INH administration causes periodic decrease of its concentration below the effective minimum inhibitory concentration (MIC), allowing MTB bacilli to develop resistance (30). The distribution of the antituberculous drug within the infected tissue is equally important. The studies of Kjellsson et al. (31) on a TB infected animal model have shown that after systemic administration the distribution of isoniazid, rifampicin and pyrazinamide in the lung tissue is uneven. The concentrations of drugs in pulmonary lesions where the pathogen is located have been markedly lower than in the surrounding lung tissue.

Hickey et al. (32) indicated the problem of dramatic increase in extremely drug-resistant TB around the world and highlighted that pulmonary administration offers the ability to deliver drug directly to the infected macrophages in the deepest part of lungs. Comparison of various dry-powders containing anti-TB drugs for inhalation presented by Pham et al. (33) indicates that the strategy of inhaled therapy is the main and the most promising alternative to traditional approach and is necessary to achieve global TB control.

The MOF-based theranostics may be an interesting alternative for the standard therapy of tuberculosis. Pulmonary route should ensure high local drug concentration (avoiding side effects of the systemic drug action). Moreover, possibility to generate contrast in magnetic resonance images should allow deposition and lung clearance monitoring (34–36) – the first tests on animals were performed in a clinical system with a clinical nebulization setup and a low inhaled dose (34). There are two reasons for deposition monitoring: optimization of the dosage form at formulation stage and optimization of therapy.

The aim of the study was initial evaluation of applicability of MOF Fe-MIL-101-NH_2_ as a theranostic carrier of antituberculous drug in terms of its functionality, i.e. drug loading, drug dissolution, MRI contrast and cytotoxic safety.

## Materials and Methods

### MOF Synthesis and Drug Incorporation

Fe-MIL-101-NH_2_ was synthesized according to the procedure reported by Bauer et al. (37). The MOF powder samples were comminuted by milling in an agate ball mill for 24 h and then sonicated for 5 min. Using a CP 130 PB (130 W, 20 kHz) ultrasonic processor (Cole-Parmer, USA), at 70% of maximum amplitude. After drying MOF was activated under vacuum at 100°C for 30 min.

Isoniazid (Sigma-Aldrich, Germany) was incorporated into the MOF matrix by mixing 1.5 mL of saturated solution of INH in DMF with 300 mg of micronized Fe-MIL-101-NH_2._ The slurry was mixed for 12 h at room temperature and the product was separated by centrifugation and washed with ethanol.

### MOF Characterisation

The characterization by X-ray powder diffraction was carried out using a Bruker AXS D8 Advance (Bruker, Germany) diffractometer in the range 1–30° 2Θ using CuKα (λ = 0.154178 nm) radiation.

Infrared (IR) spectra were measured in transmission mode using Tensor 27 FTIR spectrometer (Bruker, Germany) equipped with an MCT (Mercury-Cadmium-Telluride) detector at spectral resolution of 2 cm^−1^. Before measurement a sample was deposited on an IR-transparent silicon wafer (pure for electronic purposes) by placing its ethanol solution directly on the disc and evaporating the solvent. The wafer was placed in an IR cell with KBr windows and slowly heated under constant pumping (10^−3^ Torr) to 50°C (with the drug present) or 100°C (pure MOF).

Scanning Electron Microscopy (SEM) analysis was performed using Nova Nano SEM 200 (FEI Europe B.V.) cooperating with the Element Energy Dispersive Spectroscopy (EDS) analyzer (EDAX Inc., U.S.A.) using secondary electrons in low vacuum conditions (60 Pa). Samples of MOF without treatment, MOF after milling and MOF after milling and sonication were analyzed.

The particle size distribution of the powder samples was measured in terms of particle diameter at 50% in the cumulative distribution (Dx (38)) using laser diffraction particle size analyzer Mastersizer 3000 (Malvern Instruments Ltd., United Kingdom).

### Drug Release

The drug release study was performed in Franz cells (39). The donor compartment was separated from the acceptor compartment by a cellulose acetate filter with pore size of 0.8 μm (Sartorius, Germany) covered with 200 μL of 1% mucin solution simulating the mucus layer deposited on lung epithelium (40–43). The 5 mg of composite powder was placed in the donor compartment. The acceptor compartment was filled with 5 mL of phosphate-buffered saline (PBS) at pH 7.4 (44,45). The experiment was performed at 37°C under continuous stirring at 140 rpm. Samples of 0.4 mL were taken from the acceptor compartment at 0.5, 1, 1.5, 2, 3, 4, 5, 6, 8, 10, 24 and 48 h and were replaced with 0.4 mL of a fresh medium. A blank was carried out by evaluation of released crystalline isoniazid in quantity corresponding to the content of drug in MOF. The isoniazid concentration was analyzed in an aqueous solution using a UV-Vis spectrophotometer UV 1800 (Shimadzu, Japan) at a wavelength λ = 262.0 nm.

### Magnetic Resonance Imaging and Relaxometry

Nuclear magnetic resonance imaging and relaxometry were performed using 9.4 T MRI research system (Bruker Biospin, Germany) and TopSpin 2.0 software (Bruker Biospin, Germany). For this purpose 0.3, 1.7, 3.3, 6.7, 13.3, 26.7 and 53.3 mg/mL MOF suspensions in 2% hydroxypropylmethylcellulose (HPMC; Metolose, 90SH, 400 cP - Shin-Etsu, Japan) water solution were prepared.

T_1_ and T_2_ relaxation times were measured using Inversion Recovery and Carr Purcell Meiboom Gill (CPMG) pulse sequences, respectively – number of accumulations (NA) = 8 and dwell time (DW) = 10 μs. For T_1_ assessment separate measurements with 16 logarithmically spaced inversion time values were performed in order to sample T_1_ recovery. For CPMG sequence the signal was acquired after 16 logarithmically spaced n• TE time intervals (where TE = 0.2 ms). The data were fitted assuming monoexponential T_2_ decay (T_1_ recovery). Linear regression of R_1_ (= 1/T_1_) and R_2_ (= 1/T_2_) vs. MOF concentration in suspension was performed to obtain r_1_ and r_2_ relaxivities with intercept set at R_1_ and R_2_ values of pure polymer solution.

MR imaging was performed using Multi-Slice Multi-Echo (MSME) imaging sequence for pure 2% HPMC solution as well as for 26.7 and 53.3 mg/mL MOF suspensions in 2% HPMC solution. Two sets of images at two different repetition time (TR), i.e. 0.7 s and 3 s were obtained with following parameters: field of view (FOV) = 28 × 28 mm^2^, slice thickness = 1 mm (axial cross section), image matrix size of 256 × 256, number of echoes (NE) = 256, inter-echo time (TE) = 3.5 ms, NA = 2.

### *In Vitro* Cytotoxicity

For MOF cytotoxicity study, commercially available fibroblasts L929 (Sigma-Aldrich, Germany) were cultured in Eagle’s Minimum Essential Medium supplemented with 10% fetal bovine serum (ATCC, USA). Cells were maintained at 37°C in a humidified incubator (ThermoSci, Germany) with 5% CO_2_ until 70–80% confluence was obtained. At passage 3 the cells were detached using 0.5% trypsin-EDTA, centrifuged and suspended in the fresh growth medium. Next, the cells were seeded at density 0.5 · 10^4^ cells/200 μL in 96-well culture plates (Nunc, Denmark) and allowed to adhere. After 24 h, suspensions of milled or milled and sonicated particles of Fe-MIL-101-NH_2_ were added to the culture at two concentrations: 0.625 and 1.25 mg/mL.

Viability of L929 fibroblasts cultured in contact with MOF suspension for 24 or 72 h was determined with resazurin-based reagent PrestoBlue™ (Invitrogen, USA). Fluorescent product of the reaction was detected using POLARstar Omega microplate reader (BMG Labtech, Germany). Obtained results are presented as the mean ± standard deviation (SD) of five samples. Significant effects (*p* < 0.05) were determined using Student’s *t*-test. Cells morphology was observed under contrast phase inverted microscope CKX53 (Olympus, Japan).

## Results and Discussion

### Synthesis and Characterization of MOFs.

**Fig. 1:**
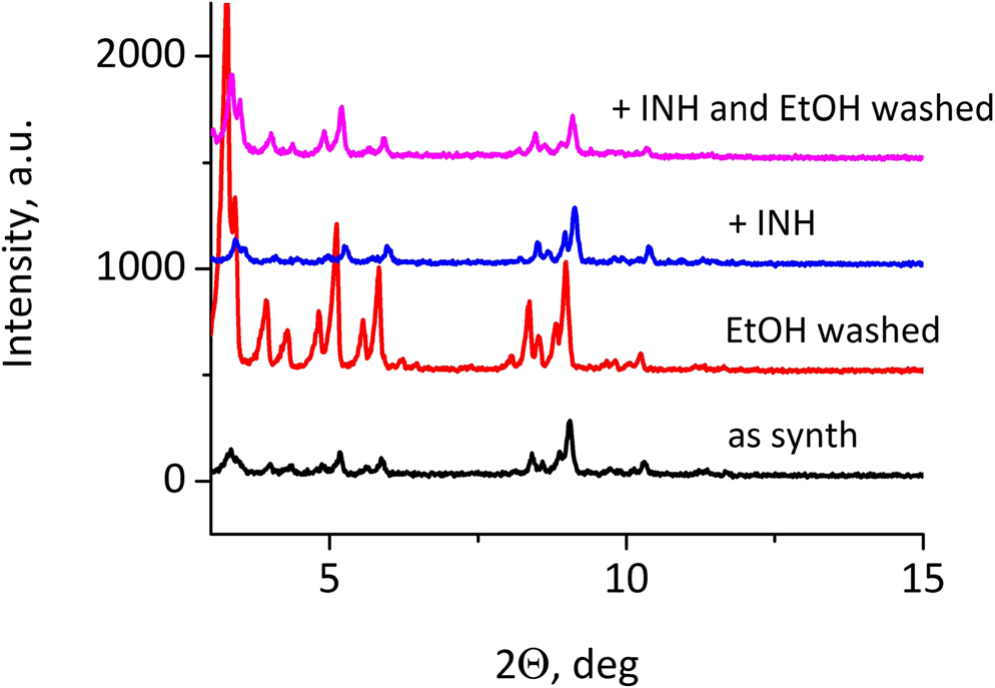
**XRD patterns of Fe-MIL-101-NH**_**2**_**:** as synthesized, washed with ethanol, after introduction of isoniazid and again washed with ethanol after isoniazid introduction (from bottom to top)

Fe-MIL-101-NH_2_ of good quality was synthesied, as confirmed by the diffractogram (XRD pattern) (Fig. 1), which showed a very good agreement with the calculated MIL-101 patterns published in the literature (46). The intensities of the reflections increased considerably after washing with ethanol of both the as-synthesized sample, containing free DMF molecules, and for the MOF sample with incorporated INH. This is most likely due to the common effect observed when micropores are emptied from the occluded guest molecules.

Iron-containing MOF compound Fe-MIL-101-NH_2_ was chosen for two reasons. First, it is one of the most stable Fe-based Metal-Organic Frameworks which has been already reported as a carrier for bioactive (47,48) or magnetic (49,50) compounds. Second, the presence of the –NH_2_ functional groups in the linker allows easy determination whether guest molecules are located inside the MOF cavities, interacting with them and changing their properties, or are only adsorbed in the intercrystalline voids.

MIL-101 has a rigid zeotype (MTN) crystal structure (50) with two types of cages. Its medium size cavities with the diameter of 2.9 nm are accessible through pentagonal windows with the opening of 1.2 nm, while large 3.4 nm cavities have hexagonal windows with the diameter of 1.6 nm (51). The 3D molecular size for INH was determined (52) using Chem Office Software 2008 as equal to 1.05 × 0.72 × 0.31 nm. The spacious MOF cavities together with relatively large apertures make carrier suitable for incorporation of bulky drug molecules.

### Particle Size

**Fig. 2:**
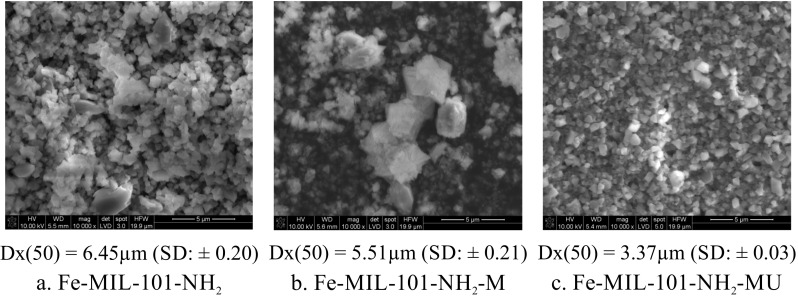
**SEM images of Fe-MIL-101-NH**_**2**_ **samples:** (**a**) without treatment, (**b**) after milling (-M), (**c**) after milling and ultrasound treatment (-MU)

Regarding particle size, according Hirschle et al. (53), MOF NPs intended to use as drug delivery systems shoud be characterized using multiple techniques – as powder and also in dispersion. They also discuss the appropriate method for obtaining the nanoparticle size that is meaningful in the context of the desired application. In our work we evaluated the impact of milling and subsequent ultrasonication on Fe-MIL-101-NH_2_ crystal size and shape. Three kinds of samples were examined: MOF without treatment (Fig. 2a), MOF after milling (Fig. 2b) and MOF after milling and ultrasonication (Fig. 2c).

For dry powders SEM imaging and measurement of particle size distribution were carried out.

Milling the samples did not change the overall shape and size of crystals, which appeared to be undamaged in SEM images (Fig. 2b). It was also not able to break crystal aggregates; thus this method cannot be used by itself for micronization of the samples. For this reason, crystals after milling were ultrasonicated, which caused breakage of the aggregates resulting in very homogeneously looking SEM images of single crystals, as shown in Fig. 2c.

The measurement of particle size distribution was performed using laser diffraction method. The Dx (38) parameter for MOF samples without treatment was 6.45 μm (SD: ± 0.20). Dx (38) decreased as a result of milling to 5.51 μm (SD: ± 0.21) and to 3.37 μm (SD: ± 0.03) after subsequent sonication. It indicates the possibility to adjust Fe-MIL-101-NH_2_ size by applying the appropriate micronization method to obtain the desired size.

### INH Incorporation

To evaluate the content of the drug inside the pores the XRD study was conducted, and no free isoniazid crystals were evident in the XRD pattern (Fig. 1). The intensities of all XRD reflections decreased markedly for the isoniazid-containing MOF, almost to the same level as observed for the as-synthesized samples filled with the solvent (DMF). Washing the drug-MOF composite with ethanol caused partial extraction of the encapsulated isoniazid, which was indicated by increased intensities of the XRD reflections. This suggests that INH molecules were located inside the pores of the MOF material.

**Fig. 3:**
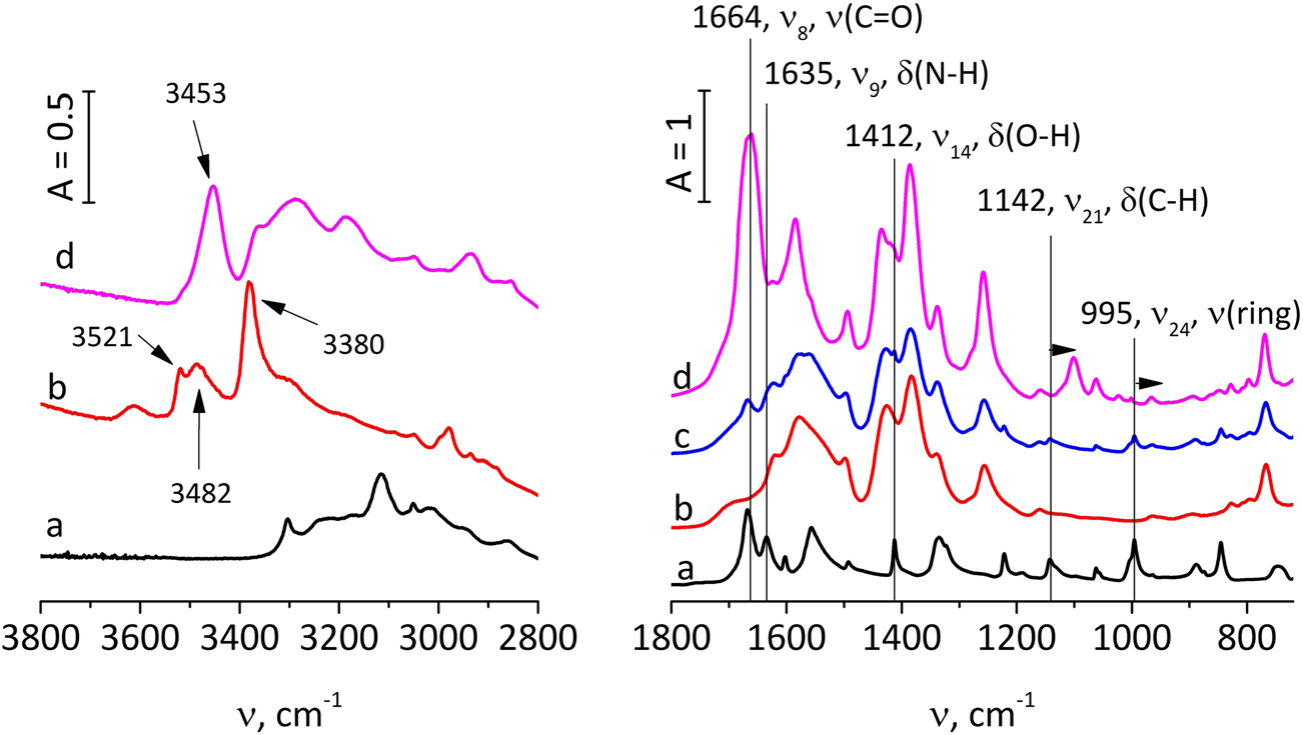
**Drug loading:** IR spectra in the N-H stretching vibration region (left) and framework vibrations (right) in transmission mode of: pure isoniazid (**a**), pure Fe-MIL-101-NH_2_ (**b**), physical mixture of Fe-MIL-101-NH_2_ with isoniazid (**c**), and isoniazid incorporated into Fe-MIL-101-NH_2_ (**d**). Arrows show the red-shift of some of the IR maxima

The mode of INH incorporation was further investigated by FTIR spectroscopy (Fig. 3). After INH introduction the spectrum was not a simple superposition of the spectra chearchterstic of the pure component of MOF and INH, again suggesting that the drug molecules were located inside the pores of Fe-MIL-101-NH_2_. In the pure MOF material the –NH_2_ groups of the structure-forming linker were characterized by two IR maxima at 3505 and 3390 cm^−1^ characteristic of ν_as_ and ν_sym_ N-H vibrations (38,54), each split into two components – one at the higher and one at the lower frequency. The lower frequency components (bands at 3485 and 3295 cm^−1^) could be due to hydrogen-bonding of the –NH_2_ moieties. This would suggest that –NH_2_ functionalities of the linker may be present both as free and the intramolecular hydrogen-bonded species even in the absence of DMF molecules. After INH introduction, the IR maxima characteristic of free –NH_2_ disappeared due to formation of new hydrogen bonds with the isoniazid molecules. The INH molecules were concluded to interact via its carbonyl groups because this particular maximum was much wider in the spectrum of the composite than in the pure, crystalline isoniazid (Fig. 3b, band at 1664 cm^−1^) and of lower frequency than expected from vibrations of the free C=O bond. The spectral characteristics of isoniazid also changed considerably. The most important changes were these in the ring breathing vibrations (Fig. 3b, band at 995 cm^−1^) – this band red-shifted (by 30 cm^−1^) and its intensity decreased. Such changes may be assigned to so-called confinement effect, resulting in constrained breathing of the aromatic ring inside the MOF pores. Similar changes, also in the 1800–1300 cm^−1^ region, were observed upon incorporation of INH into the montmorillonite and saponite clays (55). From the IR results it can be deduced that INH molecules were located inside the pores of Fe-MIL-101-NH_2_, strongly interacting by hydrogen bonding via carbonyl groups with the –NH_2_ functionalities of the organic linker. Such strong interaction may result in the slower release of the INH.

The study of total INH content by mixing in water during 12 h revealed that INH constituted 12% (SD: ± 0.8) of the composite mass. This amount was considered as 100% of the isoniazid content in the release study.

### INH Dissolution

**Fig. 4:**
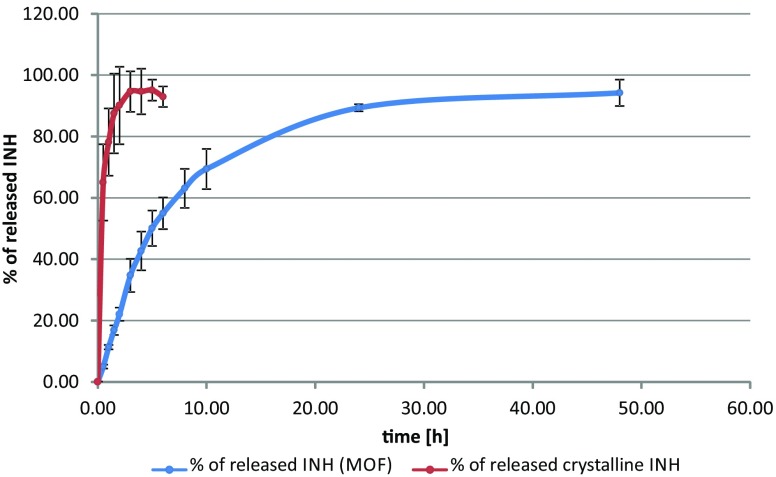
**Isoniazid dissolution:** Comparative in vitro dissolution (drug release) profile of INH from Fe-MIL-101-NH_2_ (blue) and crystalline INH (red)

After first 6 h of dissolution inside the Franz cell 55.0% (SD: ± 5.2) of the isoniazid content was released from the composite powder, it reached 89.3% (SD: ± 1.2) after 24 h and 94.2% (SD: ± 4.3) after 48 h. No burst effect was observed. Dissolution of crystalline isoniazid, not incorporated in MOF structure, was much faster and after 3 h (94.6%; SD: ± 6.6) no significant increase in the amount of released drug was observed (Fig. 4). These results allowed the conclusion that MOF can act as isoniazid carrier for extended release.

Moreover, it has been also shown, that varying NH_2_ to C_4_H_4_ linker ratio for MIL-101(Fe) it is possible to obtain continuum of guest molecules binding energy states (representing specific interaction with guest molecules) and in consequence to tune release profile. (15)

INH dissolution requires more extensive discussion. To date there are no pharmacopoeial dissolution method for inhalatory formulations and no single *in vitro* method has emerged as the ideal choice for performing dissolution tests and to estimate *in vivo* solubility in the lung fluids (56,57). The reason is that lungs have unique features that are difficult to replicate *in vitro*, such as extremely small amount of aqueous fluid and the presence of lung mucus and surfactant (56–59).

In the current study, similar setup was used as it has been previously presented, for example, by Kim et al. (39). Due to the physiology of the lung and the relatively low water content in the respiratory tract, the 5 mL Franz cells allow *in vitro* approach in comparing the drug release profiles of inhalation dry powder formulations (60,61). Highly viscous mucus is a major obstacle for particles to reach the respiratory airway (41) thus, similarly to Terzano et al. (40) in our study the mucin solution imitating mucus barrier was used.

Observation made by May et al. (62) revealed that dissolution profiles obtained in Franz cell never reached more than 90% of recovery rate which might be caused by not homogenous contact area to dissolution medium under membrane due to small air bubbles or wrinkles in the membrane. Similarly, in our study incomplete dissolution (~95%) can also be observed for both crystalline isoniazid powder and INH incorporated in MOF.

### Nuclear Magnetic Resonance Relaxometry and Magnetic Resonance Imaging

**Fig. 5:**
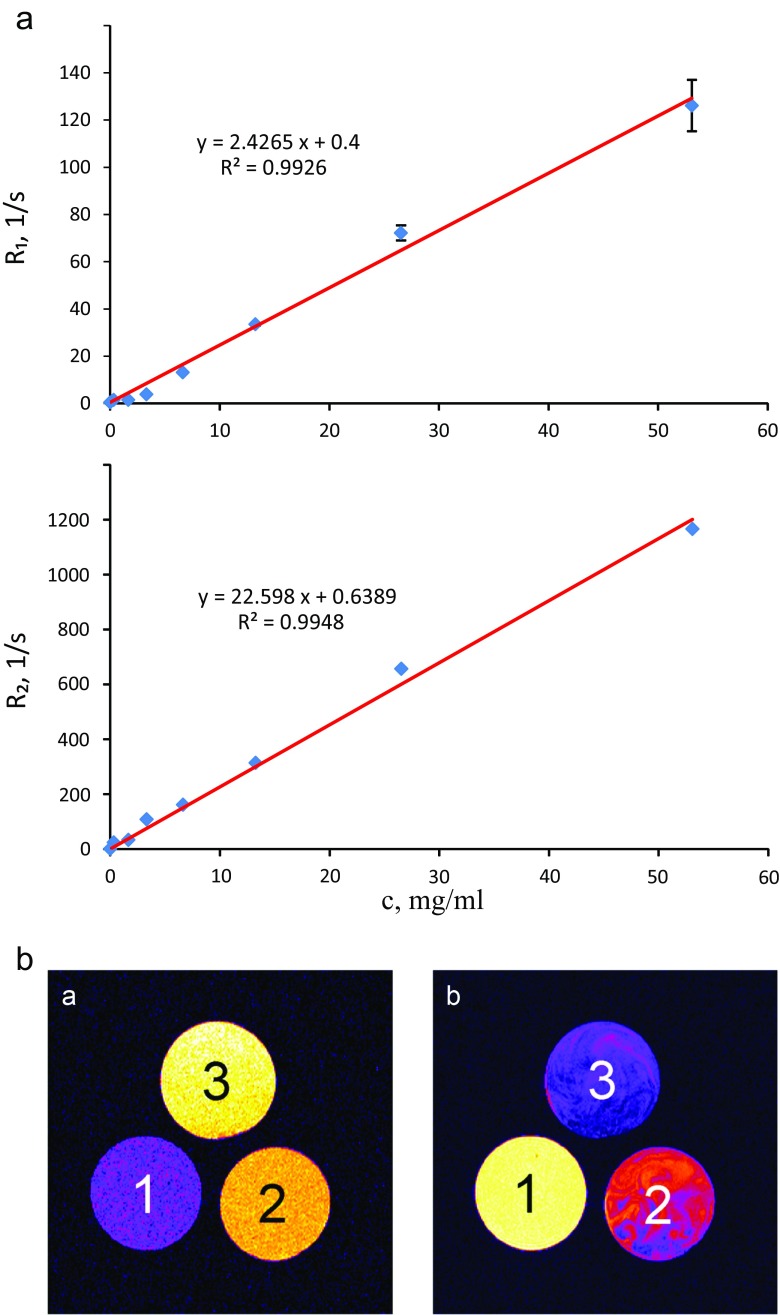
**Nuclear Magnetic Resonance Relaxometry and Magnetic Resonance Imaging:** (**a**) The quantitative linear correlation between relaxation rates (R_1_, R_2_) and concentration of Fe-MIL-101-NH_2_ in a suspension. (**b**) MR images of FeMIL-101-NH_2_ showing the examples of positive (left image) and negative (right image) contrast due to differences in imaging sequence parameters (TR = 0.7 s, TE = 3.5 ms and TR = 3 s, TE = 21 ms respectively)

The spin-lattice relaxation rates (1/T_1_ or R_1_) and spin-spin relaxation rates (1/T_2_ or R_2_) versus concentration of MOF in the suspensions are presented in Fig. 5a. The relaxation rates R_1_ and R_2_ increased linearly with the concentrations of suspended material (R^2^ = 0.9926 and R^2^ = 0.9948, respectively). The relationships between relaxation rates and concentration of Fe-MIL-101-NH_2_ in the suspensions were found to be equal to:

1 $$ {\mathrm{R}}_{1,2}={\mathrm{r}}_{1,2}\mathrm{C}+{\mathrm{i}}_{1,2} $$

Where C is the concentration of Fe-MIL-101-NH_2_ in the suspension, expressed in mg/mL. The values of relaxivity r_1_ = 2.4 (mg/mL)^−1^ s^−1^ and intercept i_1_ = 0.4 s^−1^ for T_1_ relaxation while r_2_ = 22.6 (mg/ml)^−1^ s^−1^ and intercept i_2_ = 0.64 s^−1^ for T_2_ relaxation were found, respectively. These results suggested that Fe-MIL-101-NH_2_ could be used as an effective contrast agent.

To demonstrate the possibility to use Fe-MIL-101-NH_2_ as an effective contrast agent, MR images were obtained using multi-echo pulse sequence with inter-echo time of 3.5 ms and with two different repetition times, i.e. 0.7 and 3 s. Therefore, two sets of images were obtained. Images demonstrating positive and negative contrasts were chosen from these two image sets, and are presented in Fig. 5b. Sample No. 1 was a 2% HPMC solution. Two other samples were 26.7 and 53.3 mg/ml MOF suspensions in 2% HPMC solution, i.e. samples No. 2 and 3 respectively.

When working with short repetition time of 0.7 s at the 1st echo (3.5 ms), positive contrast was obtained (Fig. 5b, left image). In this case, the image intensity for samples No. 2 and No. 3 was higher than for sample No. 1 and it increased with MOF concentration. An image obtained at the 2nd echo (echo time of 7 ms) of this image set also demonstrated positive contrast compared to the reference sample, however the difference in image intensity between samples No. 2 and No. 3 was negligible (data not presented). When working with the long repetition time of 3 s (Fig. 5b, right image) negative contrast was achieved. As an example, the image obtained at 6th echo (echo time of 21 ms) is presented. In this case, image intensity for samples No. 2 and No. 3 was lower than for reference (sample No. 1 – pure polymer solution) and it decreased with increasing MOF concentration.

Only small number of *ex vivo* and one *in vivo* study showed that regional distribution/deposition of aerosol, containing MRI contrast agents (iron oxide, Gd-DOTA) in rat lungs can be successfully monitored using MRI (34,35). The results of these studies suggest that the approach to combine drug delivery with contrast agent (theranostic) is promising for such demanding application.

#### In Vitro Viability/Cytotoxicity

**Fig. 6:**
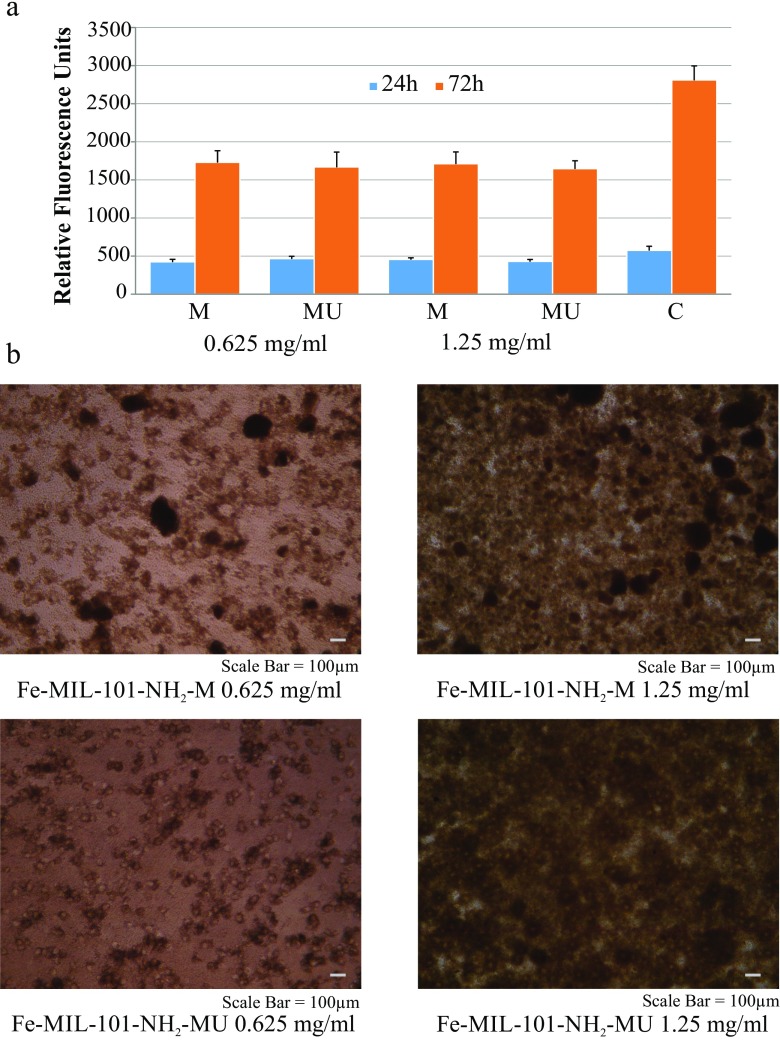
**MOF cytotoxicity:** (**a**) Dependence of fluorescence (in relative fluorescence units) on the concentration of Fe-MIL-101-NH_2_ contacted with L929 fibroblasts for 24 and 72 h. M – milled and MU – milled and ultrasonicated samples. C - control. (**b**) Photomicrographs of morphology of L929 fibroblasts after 24 h culture with addition of 0.625 mg/ml or 1.25 mg/ml of Fe-MIL-101-NH_2_ MOF. M – milled and MU – milled and ultrasonicated sample

The viability of fibroblasts cultured for 24 h with both concentration of Fe-MIL-101-NH_2_ (0.625 and 1.25 mg/mL) did not differ between samples and between samples and the control (Fig. 6). No cytotoxic effect of MOF was observed for this series. After 72 h the viability of cells cultured with MOF samples was significantly (*p* < 0.05) lower in comparison to the control, but the number of cells was still higher than observed after 24 h, which means that the addition of MOF did not inhibit their proliferation. It was also shown that micronization of MOF crystals did not influence their cytotoxicity.

The morphology of L929 fibroblasts after 24 h culture with the addition of two concentrations of milled or milled and sonicated particles of Fe-MIL-101-NH_2_ is presented in Fig. 6b. In the case of 1.25 mg/mL MOF concentration, the cells were eclipsed with the particles. MOF particles in lower concentration (0.625 mg/mL) were mostly phagocytosed and were clearly visible inside the cells. It proved that Fe-MIL-101-NH_2_ particles could release drug inside cells. Tubercle bacilli after reaching the alveoli are phagocytosed and accumulate in alveolar macrophages to form tubercles. It implies that delivery of isoniazid directly to the cell increase the effectiveness of therapy. In our study, no damage in L929 cells’ morphology (shape and appearance) after treatment was observed. Majority of cells had elongated shape, characteristic for fibroblast that proved the cells’ viability. Cytotoxicity results described above are the first results for Fe-MIL-101-NH_2_ – to the best of our knowledge such study has not been published previously. Moreover, studies on MOF materials toxicity are scarce. The benefits of MOF miniaturization, apart from their proven effectiveness in cellurar uptake, defined their *in vivo* fate and consequently, their toxicity/activity (63).

Wuttke et al. conducted cytotoxicity study for MIL-100(Fe) and MIL-101(Cr) nanoparticles with and without lipid (1,2-dioleoyl-sn-glycero-3-phosphocholine) layer on human endothelial cells (HUVEC and HMEC), alveolar epithelial cells (MLE12) and mouse alveolar macrophages (MH-S). Results revealed that both MOFs are well tolerated by endothelial cells whereas the MIL-100(Fe) with a lipid layer caused some apoptotic cell death. Alveolar epithelial cells tolerate only lipid-coated MOF at lower doses of up to 50–100 μg mL-1. Alveolar macrophages appear to be particularly sensitive to iron MOF, which cause pronounced induction of a cellular stress response.

Grall et al. (64) have recently investigated in vitro cell toxicity of Fe-MIL-100 nanoparticles and their Cr and Al analogue nanoparticles on lung (A549 and Calu-3) and hepatic (HepG2 and Hep3B) cell lines. Authors proved that pulmonary, ingestion or intravenous exposure modes were not toxic to the investigated cell lines. The examples above, reveal that the tested MOF show differential toxicity and bioresponse in different effector cells tested, which indicate their differential suitability for specific medical purposes (11). In the study by Baati et al. (65), it has been demonstrated, that high doses (220 mg/kg) of Fe-MOFs (MIL-100, MIL-88A and MIL-88B-4CH_3_) have shown no severe *in vivo* toxicity when administered intravenously to rats (65).

Similarly to previous studies performed on various members of the MIL family, the results presented in the current study revealed the low cytotoxicity of investigated Fe-MIL-101-NH_2_ material. It proves safety of Fe-MIL-101-NH_2_ as a potential drug carrier.

## Conclusions

This work shows that Fe-MIL-101-NH_2_ Metal-Organic Framework can be an effective carrier for first-line anti-tuberculosis antibiotic – isoniazid. The developed material assured sustained drug release in opposite to fast dissolution of crystalline isoniazid powder. Additionally, magnetic resonance imaging and relaxometry on phantoms of the MOF system suspended in HPMC solution proved that proposed drug delivery system based on iron-MOF can also serve as the MRI contrast agent. These two features: drug delivery and imaging properties, combined in one carrier allow to classify Fe-MIL-101-NH_2_ as theranostic agent.

According to performed in vitro cytotoxicity study the material was found to be safe. It has been observed that Fe-MIL-101-NH_2_ particles were accumulated in the cell cytoplasmic area and were able to release drug inside cells, which makes them promising drug delivery system for local TB therapy. It can be expected that local drug action accomplished this way should increase therapy effectiveness, due to direct drug delivery to the MTB bacilli locations and diminish clinically assessed side effects of traditional systemic drug administration.

Presented results are the first step in development of inhalable drug delivery system based on iron-MOF. The obtained results suggest that it will be possible to optimize flow properties of the system to assure drug loaded MOF particles to reach alveoli level. Incorporation of other anti-TB drugs into MOF structure seems to be promising to ensure multi-therapy and in consequence, prevent the development of MTB bacilli resistance. Illes et al. (22,66) indicated the applicability of MOF in multi-drug therapy which has proven to be more effective than single-drug therapies in cancer treatment and in tuberculosis therapy is even obligatory.

In the advent of feasible translation of inhalable, pulmonary deposition monitoring to human (34), the application of MOF for extendedrelease inhalable system proposes the novel strategy for delivery of standard antimycobacterial agents combined with the monitoring of their distribution within the lung tissue.

## Abbreviations

CPMG: Carr Purcell Meiboom Gill
DDS: Drug delivery systems
DMF: Dimethylformamide
EDS: Element Energy Dispersive Spectroscopy
FOV: Field of view
FTIR: Fourier-transform infrared spectroscopy
HPMC: Hydroxypropylmethylcellulose
INH: Isoniazid
IR: Infrared
MCT: Mercury-Cadmium-Telluride
MIC: Minimum inhibitory concentration
MOF: Metal organic framework
MRI: Magnetic resonance imaging
MSME: Multi-Slice Multi-Echo
MTB: Mycobacterium tuberculosis
NE: Number of echoes
NP: Nanoparticles
PBS: Phosphate-buffered saline
PDT: Photodynamic therapy
SD: Standard deviation
SEM: Scanning Electron Microscopy
TB: Tuberculosis
TE: Inter-echo time
TR: Repetition time
WHO: World Health Organization
XRD: X-ray powder diffraction
